# Quantitative real-time optical imaging of the tissue metabolic rate of oxygen consumption

**DOI:** 10.1117/1.JBO.23.3.036013

**Published:** 2018-03-24

**Authors:** Michael Ghijsen, Griffin R. Lentsch, Sylvain Gioux, Matthew Brenner, Anthony J. Durkin, Bernard Choi, Bruce J. Tromberg

**Affiliations:** a Beckman Laser Institute and Medical Clinic, Laser Microbeam and Medical Program, Irvine, California, United States; b University of California, Department of Biomedical Engineering, Irvine, California, United States; c University of Strasbourgh, ICube Laboratory, Illkirch, France; d University of California, Irvine Medical Center, Department of Medicine, Division of Pulmonology, Orange, California, United States; e University of California, Irvine Medical Center, Department of Surgery, Orange, California, United States

**Keywords:** tissue metabolism, tissue optics, scattering, absorption, speckle contrast

## Abstract

The tissue metabolic rate of oxygen consumption (${\mathrm{tMRO}}_{2}$) is a clinically relevant marker for a number of pathologies including cancer and arterial occlusive disease. We present and validate a noncontact method for quantitatively mapping ${\mathrm{tMRO}}_{2}$ over a wide, scalable field of view at 16  frames/s. We achieve this by developing a dual-wavelength, near-infrared coherent spatial frequency-domain imaging (cSFDI) system to calculate tissue optical properties (i.e., absorption, $\mu_{a}$, and reduced scattering, $\mu_{s}^{\prime }$, parameters) as well as the speckle flow index (SFI) at every pixel. Images of tissue oxy- and deoxyhemoglobin concentration ($\left[{\mathrm{HbO}}_{2}\right]$ and [HHb]) are calculated from optical properties and combined with SFI to calculate ${\mathrm{tMRO}}_{2}$. We validate the system using a series of yeast-hemoglobin tissue-simulating phantoms and conduct *in vivo* tests in humans using arterial occlusions that demonstrate sensitivity to tissue metabolic oxygen debt and its repayment. Finally, we image the impact of cyanide exposure and toxicity reversal in an *in vivo* rabbit model showing clear instances of mitochondrial uncoupling and significantly diminished ${\mathrm{tMRO}}_{2}$. We conclude that dual-wavelength cSFDI provides rapid, quantitative, wide-field mapping of ${\mathrm{tMRO}}_{2}$ that can reveal unique spatial and temporal dynamics relevant to tissue pathology and viability.

## 1 Introduction

Many disease states are known to have aberrations in tissue metabolism caused by adaptations to local cellular conditions.^1^^–^^6^ In cancer, for example, abnormal metabolism fuels rapid cellular growth while maintaining relative insensitivity to oxygen supply.^2^^,^^7^ Another prominent example is peripheral arterial disease (PAD), a silently progressive condition in which long-term atherosclerosis leads to decreased perfusion in peripheral circulation.^8^ In this situation, tissue is known to downregulate oxygen utilization in response to chronically low oxygen tension. Chronic wounds, coronary artery disease, diabetes, and neurodegenerative disorders all have similar features.^5^^,^^6^^,^^8^^,^^9^ In all of these conditions, quantitative dynamic imaging of the tissue metabolic rate of oxygen consumption (${\mathrm{tMRO}}_{2}$) over large, scalable regions of tissue could provide an approach to early diagnosis, screening, and treatment response monitoring.

Positron emission tomography (PET) is one such method that measures ${\mathrm{tMRO}}_{2}$.^10^ In this technique, intravenously administered fluorodeoxyglucose accumulates in regions of high tissue metabolism and emits two gamma rays in opposite directions when the fluoride isotope decays. These gamma rays, detected by the PET system, are used to characterize metabolic activity. Although PET has had widespread adoption in oncology and neuroimaging, it employs an exogenous, radioactive contrast agent, limiting its utility for more routine applications. Functional magnetic resonance imaging (fMRI) is another method that quantifies metabolic information by measuring the blood-oxygen-level dependent (BOLD) signal, typically in cerebral circulation.^11^ BOLD fMRI characterizes oxygen consumption by taking advantage of the different magnetic properties of oxy- and deoxyhemoglobin. Although BOLD-fMRI is a noninvasive technique that does not require exogenous contrast, measurement protocols are time consuming and rely on expensive scanners.

Optical imaging and spectroscopic methods offer a unique strategy for noninvasive and inexpensive tissue metabolic measurements. One example is diffuse optical spectroscopy (DOS), a near-infrared functional imaging technique capable of measuring hemodynamics and biochemical composition in centimeter-thick tissues^12^^–^^14^ and diffuse correlation spectroscopy (DCS), a complementary optical technique that measures relative blood flow.^15^^,^^16^ Together DOS and DCS are capable of characterizing tissue metabolic activity.^17^ However, DOS–DCS is typically performed using contact probes and point-by-point measurements. This impacts their utility in compromised tissues, such as wounds, where contact may not be an option and in heterogeneous regions where single point measurements are insufficient.

Spatial frequency-domain imaging (SFDI), a noncontact wide-field imaging technique capable of quantitatively mapping tissue optical properties, addresses these shortcomings.^18^^,^^19^ Similar to DOS, SFDI does not directly measure perfusion and is therefore incapable of independently determining the tissue metabolic rate of oxygen consumption in the presence of blood flow. Laser speckle imaging (LSI) is a related wide-field technique that uses coherent light sources to generate spatial maps of relative tissue perfusion.^20^^,^^21^ Together, SFDI and LSI are capable of measuring tissue oxy- and deoxyhemoglobin concentration and relative blood flow, respectively, therefore enabling ${\mathrm{tMRO}}_{2}$ imaging.

Coherent spatial frequency-domain imaging (cSFDI) is a camera-based wide-field imaging technique that combines LSI and SFDI and is capable of recovering tissue absorption and reduced scattering coefficients ($\mu_{a}$ and $\mu_{s}^{\prime }$) and speckle contrast in superficial tissues up to ∼5  mm in depth.^18^^,^^19^^,^^22^^–^^26^ By measuring $\mu_{a}$ at multiple discrete wavelengths, concentrations of tissue chromophore such as oxy- and deoxyhemoglobin ($\left[{\mathrm{HbO}}_{2}\right]$ and [HHb], respectively) can accurately be recovered. Speckle contrast can then be converted into a measurement of relative blood flow^21^^,^^27^^–^^32^ referred to here as speckle flow index (SFI). By synthesizing measurements of blood flow and chromophore concentrations, it is possible to invoke Fick’s principle and extract ${\mathrm{tMRO}}_{2}$, as previously performed with MRI^33^ and optical imaging.^34^ However, one practical limitation in applying this technique has been the large amount of data needed to recover ${\mathrm{tMRO}}_{2}$ at a single time point.^35^^–^^37^ This limits continuous acquisition speed and may result in loss of dynamic metabolic information.

In recent work, we significantly increased cSFDI data acquisition speed, thereby making it practical for quantitative real-time *in vivo* tissue measurements.^36^^,^^38^ This was accomplished by adapting Fourier-based demodulation techniques allowing for the acquisition of $\mu_{a}$, $\mu_{s}^{\prime }$, and SFI from a single snapshot, ameliorating a data acquisition bottleneck. In addition, this advance allowed for significantly simplified instrumentation by replacing complex hardware with computational analysis. Although this system could extract $\mu_{a}$, $\mu_{s}^{\prime }$, and SFI in real time, it contained only one wavelength and was therefore unable to measure chromophore concentration and oxygen metabolism.

In this work, we present and validate a high-speed, dual-wavelength cSFDI system capable of imaging blood flow coregistered with $\left[{\mathrm{HbO}}_{2}\right]$ and [HHb] at 16  frames/s over wide tissue regions (15  cm×15  cm) and use this data to calculate ${\mathrm{tMRO}}_{2}$. We validate dynamic chromophore measurements using yeast-hemoglobin-Intralipid phantoms in which bovine hemoglobin is progressively desaturated using baker’s yeast. In addition, we show sensitivity to chromophore concentration using a hemoglobin titration phantom in which portions of bovine red cells are added in series. We then apply this system to a 5-min arterial occlusion protocol and demonstrate the ability to measure changes in metabolism associated with ischemia and reperfusion in human subjects. Finally, we apply cSFDI to a rabbit model of cyanide poisoning and show sensitivity to mitochondrial uncoupling as well as the reversal of cyanide toxicity with hexachloroplatinate, a rapidly acting antidote.

## 2 Materials and Methods

### 2.1 Instrumentation

**Fig. 1:**
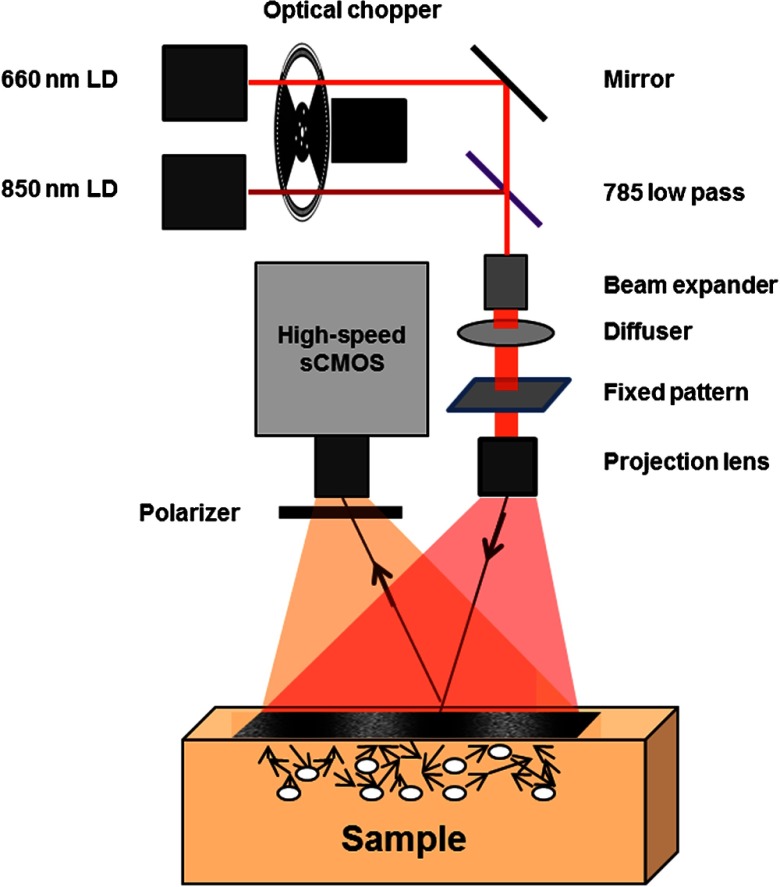
Schematic of dual-wavelength cSFDI. Light from two coherent light sources is spatially modulated at $0.2\;{\mathrm{mm}}^{-1}$ and projected onto a diffuse medium. Remitted light from the sample is collected using a high-speed sCMOS detector.

Figure 1 is a schematic of the prototype cSFDI system. Two laser diodes were used as light sources: a 660-nm 150-mW laser diode (HL6545MG, Opnext) and an 852-nm 150-mW laser diode (L852P150, ThorLabs). The current and temperature were stabilized using combined diode driver and TEC controllers (ITC4000, ThorLabs) to ensure stabilized power and speckle properties. Both diodes were mounted in thermoelectrically cooled diode mounts (LDM21, ThorLabs) and collimated using aspheric lenses. The beam path was combined using a 785-nm hot mirror (FM01 ThorLabs) and two broadband dielectric mirrors for walking the beams into the correct position. An optical chopper (MC2000, ThorLabs) was used to multiplex between the two light sources. The laser beams were widened using a custom-built beam expander, transmitted through a ground glass diffuser, and condensed onto a sinusoidal-patterned slide (SF-4.0-80-TM-G, Applied Image). The illumination spatial frequency was $0.2\;{\mathrm{mm}}^{-1}$, the working distance was 30 cm, and the field of view (FOV) was 15  cm×15  cm. The pattern was projected onto the sample using a 16-mm fixed focal length lens with VIS-NIR coating (#67-714, Edmund Optics). The sample was imaged using a high-speed scientific CMOS camera (Hamamatsu Photonics, Orca Flash 4.0) with a wire mesh polarizer (250- to 4000-nm WP25M-UB, ThorLabs Inc.) to suppress specular reflections.

### 2.2 Data and Image Processing

Raw images were processed for SFI, $\left[{\mathrm{HbO}}_{2}\right]$, and [HHb] as outlined in Ghijsen et al.^38^ and Cuccia et al.^18^^,^^19^ Single raw images were used to simultaneously demodulate two spatial frequencies using algorithms originally described in Vervandier and Gioux.^36^ In this technique, a Fourier transform was applied line-by-line to raw data to calculate a spatial frequency spectrum of each column vector within a given raw image. The frequency-domain data were then subdivided into DC and AC component spectra using top-hat window functions. The DC and AC demodulated images were then computed by performing line-by-line inverse Fourier transforms. Compared with two-frequency, three-phase SFDI, this decreased by a factor of 6 the amount of raw data needed to decouple $\mu_{a}$ and $\mu_{s}^{\prime }$, thus allowing for real-time imaging.

Data were calibrated using a phantom with known optical properties^39^ to obtain the diffuse reflectance.^18^ Optical properties at each wavelength were recovered pixel-by-pixel from the DC and AC reflectance using a Levenberg–Marquardt fit of experimental data to a light-transport model. Concentrations of ${\mathrm{HbO}}_{2}$ and HHb were obtained using $\mu_{a}$ at 660 and 852 nm along with chromophore extinction coefficient spectra as described in Mazhar et al.^25^ Using the same data, a 7-pixel×7-pixel sliding window filter was used to calculate the local standard deviation σ(I) and mean intensity ⟨I⟩ and then was used to compute the speckle contrast K=σ/⟨I⟩.^38^ SFI was calculated from speckle contrast using the following equation: $\mathrm{SFI}=1/(2TK^{2})$, where T is the integration time (10 ms in our studies) and K is the speckle contrast.^31^

Time-series data were obtained by averaging over regions of interest (ROI) within the processed data. Uncertainties were calculated by obtaining the standard deviation over these ROIs. Unless stated otherwise, each ROI was 80  pixels×80  pixels. The camera was run at 32  frames/s with an exposure time of 20 ms and aperture of f/2.6. In past work, we demonstrated that these settings optimized the tradeoff between speed and accuracy.^38^ The center 1024×1024  pixels (out of the available 2048×2048) were used to reduce the necessary storage space since each experiment required hundreds of gigabytes.

### 2.3 Yeast-Hemoglobin-Intralipid Phantoms

**Fig. 2:**
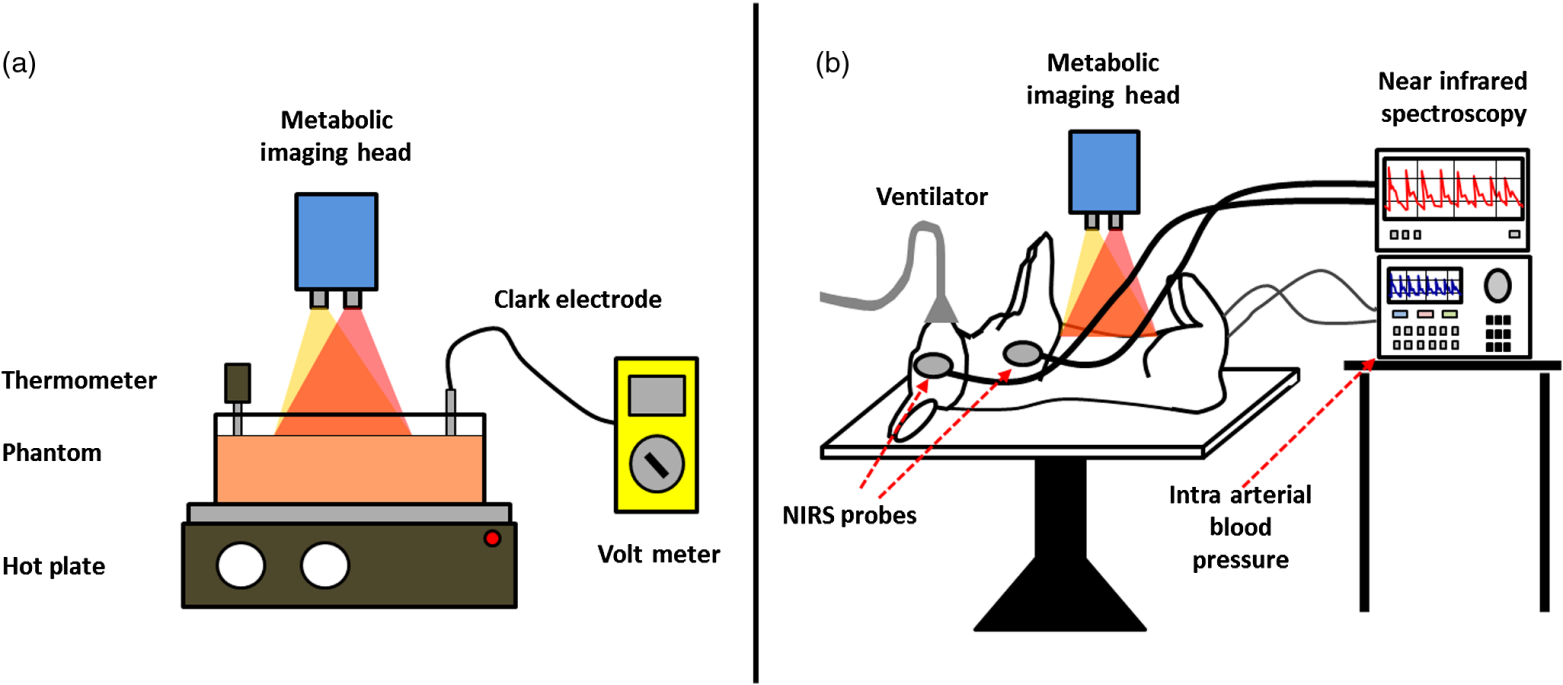
Experimental setup. (a) Yeast-hemoglobin and hemoglobin titration experiment. cSFDI is placed over a container holding the liquid phantom contents. A thermometer and Clark electrode are used to monitor the temperature and partial pressure of oxygen, respectively. A hot plate underneath the glass container keeps the phantom at 37 deg and propels a magnetic stir bar within the phantom to keep the contents well mixed. A volt meter is used to monitor the Clark electrode. (b) Rabbit-cyanide experiment. The rabbit is placed supine on the operating table. cSFDI is placed over the lower abdomen. A ventilator is used sustain the anesthetized rabbit. Two NIRS probes are placed over brain and muscle regions to monitor saturation. Intravascular lines are inserted into the femoral artery and veins to monitor blood pressure and blood gases.

A yeast-hemoglobin phantom was used to validate the ability of cSFDI to measure oxygen saturation. Yeast-hemoglobin phantoms are liquid phantoms that imitate tissue properties using Bovine hemoglobin as the active absorbing agent and Intralipid as the active scattering agent.^40^^,^^41^ Figure 2(a) shows the experimental setup for the yeast-hemoglobin phantom. A 15-cm×15-cm Pyrex dish was used to hold the contents of the liquid phantom. A thermometer and Clark-electrode monitored the temperature and partial pressure of oxygen, respectively. Bovine whole blood was centrifuged and washed with phosphate buffered saline to produce packed red cells. About 50 mL of 20% Intralipid, 934 mL of 1% phosphate buffered saline, and 16 mL of packed bovine red blood cells were combined in the Pyrex dish. The contents were then heated to 37°C using a hot plate. A magnetic stir bar was used to keep the contents well mixed. The phantom was given time to equilibrate with the ambient oxygen tension. Next, baseline measurements were taken using both the Clark electrode and cSFDI. Yeast was then dissolved in a small portion of warm water and added to the liquid phantom. Measurements were taken continuously with cSFDI and the Clark electrode until the partial pressure of oxygen reached 0 mm Hg.

### 2.4 Hemoglobin Titration Phantoms

A hemoglobin titration phantom was used to demonstrate the capability of cSFDI to measure hemoglobin concentration compared with a benchmarked system. The phantom was prepared similarly to the previous experiment. About 934 mL of phosphate buffered saline was mixed together with 50 mL of 20% Intralipid (final concentration of 1%). During this procedure, five equal preparations of bovine packed red cells were added to the liquid phantom in series such that the concentration of hemoglobin increased in approximately equal amounts. The OxImager Rs (Modulated Imaging, Inc., Irvine, California), a validated commercial SFDI system, was used for comparison. The hemoglobin titration phantom was measured before (Intralipid and PBS only) and after serial additions of blood portions using both cSFDI and the OxImager Rs. No yeast was added to deoxygenate the hemoglobin, and a Clark electrode was not used to monitor oxygen tension. After processing the data, baseline chromophore concentrations were subtracted from cSFDI’s chromophore concentrations to correct for the contribution of Intralipid to the absorption spectrum.

### 2.5 Five-Minute Arterial Occlusion Experiment

A 5-min arterial occlusion was performed on a healthy 29-year-old male subject’s left hand while being imaged with cSFDI. During this imaging procedure, the dorsal left hand was imaged while a full arterial occlusion was applied to the brachial artery using a pneumatic cuff capable of near-instantaneous inflation to 210 mm Hg (E20 Rapid Cuff Inflator, Hokanson Inc.). The duration of imaging consisted of 3 min of baseline (no occlusion), followed by 5 min of arterial occlusion, and then 3 min of postrelease. Two-dimensional (2-D) maps of $\left[{\mathrm{HbO}}_{2}\right]$, [HHb], and SFI (both wavelengths) were reconstructed for each time point for a total of 10,560 time points (11  min× 60  s/min×16  frames/s). These data were, in turn, used to produce ${\mathrm{tMRO}}_{2}$ maps. Time-series data were then obtained from ROIs and used to analyze physiology of the underlying tissue. The study was carried out under a UC Irvine IRB-approved protocol, and informed consent was obtained from the subject (HS #2011-8370).

### 2.6 Calculation of Metabolism

Indices of tissue metabolism were extracted from the 5-min arterial occlusion data. This process was done in two parts: steady state and occluded state. In the steady state, blood freely flows into and out of the tissue compartment of interest; $\left[{\mathrm{HbO}}_{2}\right]$ and [HHb] remain constant. In this situation, Fick’s principle can be invoked to relate the concentration of arterial oxygen (${\left[O_{2}\right]}_{a}$), blood flow (BF), oxygen extraction fraction (OEF), and oxygen consumption (${\mathrm{tMRO}}_{2}$)^33^

$${\mathrm{tMRO}}_{2}=(\mathrm{OEF})(\mathrm{BF})({\left[O_{2}\right]}_{a}).$$ (1)

OEF is the fraction of oxygen removed from arterial hemoglobin. In the context of this work, BF is blood flow normalized to a volume of tissue and ultimately contains units of inverse seconds ($s^{-1}$). Finally, ${\left[O_{2}\right]}_{a}$ is the molar concentration of arterial oxygen and has units of millimoles per liter. This means that ${\mathrm{tMRO}}_{2}$ has units of millimoles per liter per second. It should be noted that this model assumes 100% arterial oxygen saturation.

OEF and ${\left[O_{2}\right]}_{a}$ can be written in terms of deoxyhemoglobin concentration

$$(\mathrm{OEF})({\left[O_{2}\right]}_{a})=4[\mathrm{HHb}],$$ (2)

where [HHb] refers to the tissue concentration of deoxyhemoglobin. Additionally, normalized tissue blood flow can be written in terms of SFI

(BF)=(α)(SFI). (3)

In this equation, α is a constant that relates SFI with absolute blood flow. This is necessary because even though SFI is linearly correlated with flow, it is not an absolute measurement of flow. In these equations, SFI has also been corrected for biological zero by subtracting the SFI calculated during the arterial occlusion. Biological zero is defined as the nonzero SFI measurement in the absence of arterial flow due to Brownian motion and other possible forms of residual flow.^42^ Inserting Eqs. (2) and (3) into Eq. (1)

$${\mathrm{tMRO}}_{2}=(\alpha )(\mathrm{SFI})(4[\mathrm{HHb}]).$$ (4)

Equation (4) is the calculation of the tissue metabolic rate of oxygen consumption at steady state written in terms of measurements that can be obtained using high-speed cSFDI.

During the occluded state, blood flow is clamped off and no blood enters or leaves the tissue compartment. In this setting, ${\mathrm{tMRO}}_{2}$ can be calculated from the rate at which $\left[{\mathrm{HbO}}_{2}\right]$ is converted to [HHb]. From Cheatle et al.^43^

$${\mathrm{tMRO}}_{2}=4\frac{d([\mathrm{HHb}])}{dt}.$$ (5)

In this expression, ${\mathrm{tMRO}}_{2}$ is related to the first time-derivative of [HHb]. The factor of 4 is due to the fact that there are four oxygen molecules per hemoglobin tetramer.

Unlike Eq. (4), Eq. (5) provides a measurement of absolute metabolism; in Eq. (4) alpha is typically an unknown constant. It is possible to calculate alpha if one has *a priori* knowledge of metabolism at a certain time point. The arterial occlusion protocol provides such information. During this protocol, alpha can be calculated assuming the boundary condition that metabolism at the beginning of the occlusion is continuous with metabolism prior to the onset of the occlusion. This assumption is valid since at the onset of the occlusion the blood contained within the compartment is still rich with oxygen. Intuitively, this loses validity as the occlusion progresses and oxygen is depleted. Using the aforementioned boundary condition, Eqs. (4) and (5) can be set equal to one another

$$\alpha ({\mathrm{SFI}}_{\mathrm{bl}})(4{\left[\mathrm{HHb}\right]}_{\mathrm{bl}})=4\frac{d({\left[\mathrm{HHb}\right]}_{0})}{dt}.$$ (6)

In this expression, ${\mathrm{SFI}}_{\mathrm{bl}}$ refers to average SFI at baseline, ${\left[\mathrm{HHb}\right]}_{\mathrm{bl}}$ refers to average tissue deoxyhemoglobin concentration at baseline, and ${\left[\mathrm{HHb}\right]}_{0}$ refers to the tissue deoxyhemoglobin concentration at the beginning of the occlusion. Solving for alpha

$$\alpha =\frac{d({\left[\mathrm{HHb}\right]}_{0})}{dt}/\{({\mathrm{SFI}}_{\mathrm{bl}})({\left[\mathrm{HHb}\right]}_{\mathrm{bl}})\}.$$ (7)

This term can be reinserted into Eq. (4) to calculate the absolute ${\mathrm{tMRO}}_{2}$ following the release of the occlusion and was used to do so in this work.

For clarification, it is necessary to calculate α because it is not possible to directly determine ${\mathrm{tMRO}}_{2}$ in the presence of blood flow. This is because SFI is not an absolute metric of perfusion. Instead, ${\mathrm{tMRO}}_{2}$ is calculated during arterial occlusion when flow is zero, and then α is obtained to scale metabolism in the nonoccluded state. This is possible because SFI correlates linearly with flow.

### 2.7 Cyanide Rabbit Experiment

cSFDI was applied to a previously validated animal model in which a rabbit was infused with cyanide for a period of time and then given an antidote (hexachloroplatinate) to reverse the poisoning.^44^^,^^45^ This experiment was performed to demonstrate the ability of cSFDI to characterize metabolic changes due to cyanide exposure. Cyanide is a potent mitochondrial uncoupling agent that inhibits cytochrome c oxidase, decreasing oxygen consumption on the cellular level.^44^

A New Zealand white rabbit was anesthetized with ketamine and xylazine, intubated and placed on mechanical ventilation with 100% oxygen, and positioned supine as shown in Fig. 2. Invasive arterial and venous lines were placed in the left femoral artery and vein and used throughout the procedure to monitor blood gases. The cSFDI imaging head was positioned over the rabbit’s lower right abdomen contralateral to the intravascular lines. The fur in the imaging area was removed using electric clippers and chemical hair remover. Before the procedure began, baseline measurements of cSFDI, arterial blood gas, systemic pressure, heart-rate, and oxygen saturation were acquired. cSFDI was then used to monitor the animal through the entire experiment. Following baseline, the rabbit received a constant infusion of sodium cyanide at rate of 1-mL/min for 55 min. The infusion had a concentration of 10-mg sodium cyanide per 50 mL of normal saline. After the infusion was completed, the antidote (120-mg hexachloroplatinate in 120-μL dimethylsulfoxide and phosphate buffered saline) was administered. The animal was monitored for an additional hour following the antidote. At the end of the experiment, the rabbit was euthanized with pentobarbital. Data obtained with cSFDI were processed providing chromophores and SFI. The relative tissue metabolic rate of oxygen consumption (${\mathrm{rMRO}}_{2}$) was calculated using SFI and deoxyhemoglobin. This study was performed under Institutional Laboratory Animal Care and Use Committee protocol number 2000-2218.

## 3 Results

### 3.1 Yeast-Hemoglobin Experiment

**Fig. 3:**
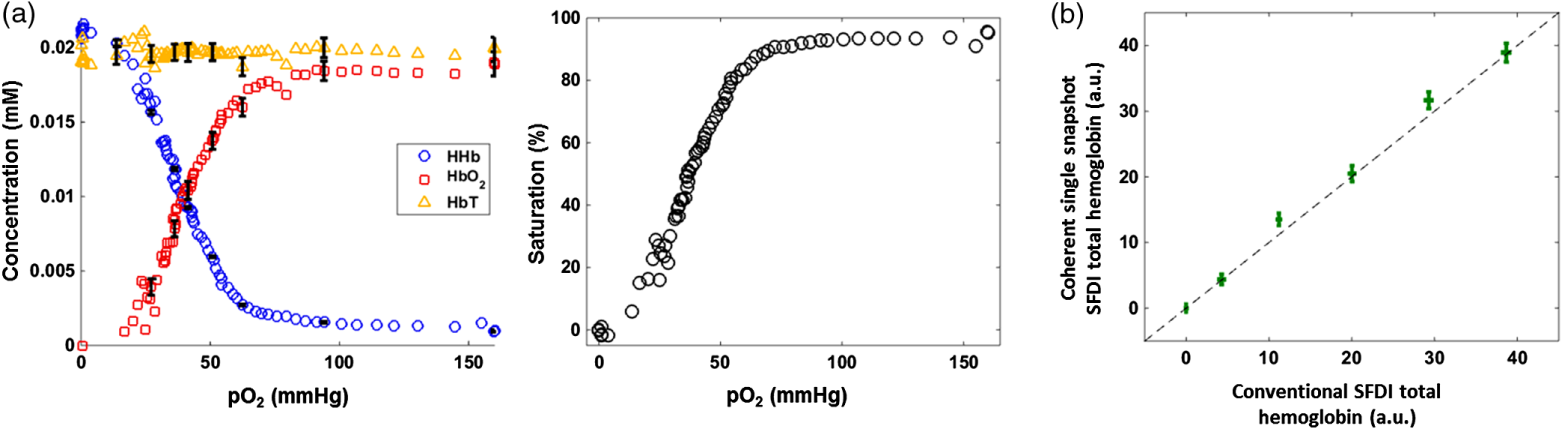
Yeast-hemoglobin and hemoglobin titration results. (a) Yeast-hemoglobin phantom results. The left panel shows blood saturation against partial pressure of oxygen. The right panel shows chromophore concentrations of deoxyhemoglobin, oxyhemoglobin, and total hemoglobin (D, O, and T, respectively). The error bars represent the standard deviation over the 80  pixel×80  pixel ROI taken from a single frame. (b) Hemoglobin titration results. The graph shows total hemoglobin concentration measured using dual-wavelength cSFDI versus total hemoglobin concentration measured using standard commercial SFDI on the x-axis. These two-dimensional error bars represent the standard deviation in each measurement over an 80  pixel×80  pixel ROI taken from a single frame of raw data. The black dotted line is a reference line of equality.

Figure 3(a) shows the chromophore concentrations (left) and saturation (right) of the yeast-hemoglobin phantom obtained from a 1-cm×1-cm ROI within the imaging field. The x-axis in both plots represents the partial pressure of oxygen determined using a Clark oxygen electrode. The plot of chromophores shows that total hemoglobin remains constant throughout the experiment. It also shows that at 0 mm Hg partial oxygen pressure, $\left[{\mathrm{HbO}}_{2}\right]$ is roughly 0 mM. As partial pressure increases to 160 mm Hg (ambient oxygen pressure), $\left[{\mathrm{HbO}}_{2}\right]$ increases to approximately the concentration of total hemoglobin, following a sigmoidal (s-shaped) curve characteristic of the hemoglobin desaturation. [HHb] follows the opposite trend. At 0 mm Hg partial pressure of oxygen, the mixture is completely composed of deoxyhemoglobin as evidenced by the fact that [HbT] and [HHb] are approximately equal. As the partial pressure of oxygen increases, [HHb] decreases along a sigmoidal curve until it settles at a concentration of about 0 mM. The error bars in the chromophore plot show the spatial variation contained within the region. Since the yeast-hemoglobin phantom is practically homogeneous, these intervals closely approximate the random system error with respect to chromophore concentration. With this in mind, the standard deviation in the random error taken from the [HbT] is 3%. The saturation plot on the right of Fig. 3(a) demonstrates that cSFDI is sensitive to the full range of saturation values, i.e., that it can detect 0% to 100% saturation. The curve also has the sigmoidal shape characteristic of hemoglobin.

### 3.2 Intralipid Hemoglobin Titration Experiment

Figure 3(b) shows the results from the hemoglobin titration experiment. The y-axis shows total hemoglobin concentration obtained with cSFDI, and the x-axis shows results obtained using a validated commercial SFDI device (OxImager Rs, Modulated Imaging, Inc., Irvine, California). Both axes are in arbitrary units due to the fact that the OxImager Rs uses proprietary software that does not specify units. These data demonstrate response linearity in terms of total hemoglobin recovery when compared with a standard commercial system. The accuracy of both technologies (SFDI and cSFDI) in terms of chromophore quantitation has been validated at length in previous work.^18^^,^^38^ This experiment is only intended to demonstrate linearity between the two instruments.

### 3.3 Five-Minute Arterial Occlusion Experiment

**Fig. 4:**
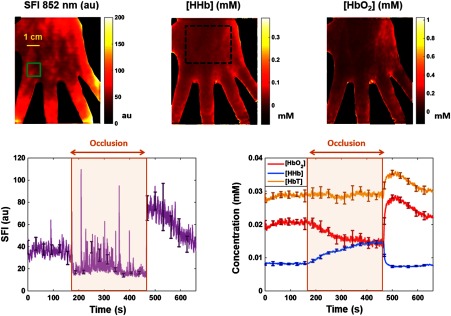
Arterial occlusion results. From left to right, the top row of images shows baseline (t=0  s) maps of SFI, deoxyhemoglobin, and oxyhemoglobin, respectively. Speckle flow is in arbitrary units while concentrations are millimoles per liter. The bottom row shows time-series data taken from a 1-cm×1-cm ROI outlined in green in the SFI image. The left graph shows SFI versus time, while the right graph shows chromophore concentration versus time. The left and right arrows in each graph show the beginning and end of the occlusion, respectively. The [HHb] image contains a black, dotted region used to calculate parameter means and variations.

Figure 4 shows results from the arterial occlusion experiment. From left to right, the top three images show maps obtained during baseline (t=0  s) of SFI at 852 nm, [HHb], and $\left[{\mathrm{HbO}}_{2}\right]$. SFI is in arbitrary units, and chromophore concentrations are in mM/L. The green rectangle in the SFI image defines the 1-cm×1-cm ROI from which the time-series data were obtained. The bottom-left plot shows SFI at 852 nm in arbitrary units versus time in seconds. The brown shaded region outlines the duration of the arterial occlusion; the period before the shaded region is the baseline and the period after is the postrelease. The error bars overlaid on the SFI plot denote the standard deviation of the values contained within the ROI. The ROI is 80  pixels×80  pixels taken from a single frame of data, i.e., multiple frames were not averaged. It should be noted that these margins not only are due to random instrument error but also take into account spatial variations of tissue properties within the ROI. On average, the standard deviation taken from the ROI in total hemoglobin displayed in Fig. 4 is 4%, slightly higher than the instrument error of 3%. At the onset of the occlusion (t=180  s), an instantaneous decrease in the SFI data is clearly observable. Likewise, upon release of the occlusion (t=480  s), there is an instantaneous increase that overshoots baseline values. The average SFI at baseline is 36 arbitrary units (au), and the average of the first 30 s postrelease is 74. During the occlusion, the SFI drops to an average of 21, referred to as biological zero.^46^ There are pulsatile spikes throughout the occlusion due to blood movement caused by muscle contractions from ischemic cramping. Subtracting biological zero from baseline and postrelease averages, these data show that perfusion within this ROI is 340% higher during the first 30 s postrelease than at baseline, demonstrating sensitivity to postocclusive reactive hyperemia.

The plot on the bottom-right of Fig. 4 shows the time-series [HbT], $\left[{\mathrm{HbO}}_{2}\right]$, and [HHb] in yellow, red, and blue, respectively. Once again, the brown shaded region outlines the arterial occlusion, with baseline and postrelease coming before and after, respectively. The error bars overlaid on the chromophore plots denote the standard deviation of the ROI due to combined instrument and biological variation. [HbT] remains constant throughout the baseline and occlusion periods. $\left[{\mathrm{HbO}}_{2}\right]$ on the other hand decreases during the occlusion while [HHb] increases; both are the results of oxygen extraction. Following the release, there is a spike in [HbT] and $\left[{\mathrm{HbO}}_{2}\right]$ caused by reactive hyperemia. [HHb] decreases as it is washed out of the tissue compartment by fresh blood.

Within the large region corresponding to the black, dotted line in the [HHb] image, the mean $\left[{\mathrm{HbO}}_{2}\right]$ is 0.07 mM and the relative standard deviation (RSD) is 75%. In the same region, the average [HHb] and SFI were 0.025 mM and 59 au, respectively. The RSD values for [HHb] and SFI were 64% and 42%, respectively. In previous work, we demonstrated using phantom studies that cSDFI-derived optical properties (and material composition) vary by 3% over a large (15  cm×15  cm) FOV. As a result, the more substantial variations seen in this work represent the heterogeneous nature of tissue, where a predominantly capillary-irrigated region has properties that differ from a region with an underlying vein. Time-series data were extracted from the smaller 80-pixel×80-pixel ROI to improve sensitivity to dynamic tissue-level changes. This is because spatial variation and differential temporal dynamics across the tissue lead to diminished signal quality when large ROIs are selected; the changes in one region are confounded by those in another. See Appendix for a description of the video included in the online component of the article.

### 3.4 Absolute and Relative Metabolism

**Fig. 5:**
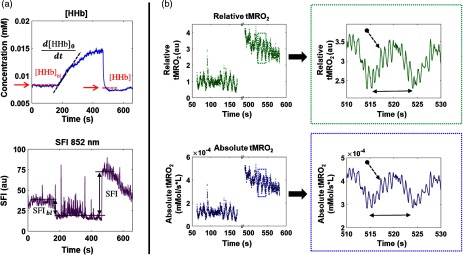
Metabolic calculation. Panel (a) contains the cSFDI parameters used to calculate absolute and relative tissue metabolic rates of oxygen consumption (${\mathrm{tMRO}}_{2}$). The top plot in panel (a) shows deoxyhemoglobin concentration and the bottom plot shows SFI at 852 nm throughout the arterial occlusion protocol. Panel (b) contains relative and absolute ${\mathrm{tMRO}}_{2}$. The two plots in green along the top contain relative metabolism and the two plots along the bottom contain absolute metabolism. The plots on the left contain the baseline and postocclusion time-series data and are separated by a break in the x-axis. The plots on the right of panel (b) contain an expanded view of a 20 s period (510 to 530 s) obtained from the dotted rectangles in the plots on the left.

Figure 5(a) shows the synthesis of absolute and relative metabolism from SFI and [HHb]. The top plot presents time-series [HHb] obtained from a single ROI. The black dotted line is a linear fit of the slope used to calculate ${\mathrm{tMRO}}_{2}$ via Eq. (5). The red dotted line on either side of the slope depicts the average [HHb] before and after the occlusion. Average baseline [HHb], denoted in the figure as ${\left[\mathrm{HHb}\right]}_{\mathrm{bl}}$, was inserted into Eq. (7). Average [HHb] postrelease, denoted in the figure as [HHb], was inserted into Eq. (4). The bottom plot in Fig. 3(a) shows SFI at 852 nm. The black double-sided arrows represent baseline SFI (left) and postrelease SFI (right). The biological zero value, defined as SFI during zero blood flow and depicted in the figure as the solid black line during the occlusion, is subtracted from the pre- and postocclusion measurements to account for this offset. Baseline SFI (${\mathrm{SFI}}_{\mathrm{bl}}$) is inserted into Eq. (7) to calculate α. Postrelease SFI (denoted SFI) is inserted into Eq. (4). Absolute and relative metabolic rates of oxygen consumption were then determined by inserting the extracted parameters derived pictorially in Fig. 5(a) into Eqs. (4), (5), and (7).

Figure 5(b) shows the relative and absolute metabolic rates from the preocclusion and postrelease periods. The two intervals are separated by a break in the x-axis, depicted by the two parallel diagonal lines intersecting the top and bottom horizontal axes. The overall trend is depicted in the top-left plot of panel b, showing relative ${\mathrm{tMRO}}_{2}$ in arbitrary units. At baseline (from approximately t=50 to 150 s), there is no general trend. However, metabolism fluctuates sinusoidally with an amplitude ∼50% of the DC-offset. During the postrelease phase (t=500 to 600 s), relative ${\mathrm{tMRO}}_{2}$ starts out at ∼4 au and gradually decreases back to baseline. Oscillatory fluctuations are simultaneously observed on top of this trend. The bottom-left plot shows absolute ${\mathrm{tMRO}}_{2}$ in units of millimoles per liter per second. Relative ${\mathrm{tMRO}}_{2}$ is calculated by dividing absolute ${\mathrm{tMRO}}_{2}$ at a given time point during postrelease by baseline ${\mathrm{tMRO}}_{2}$.

The top-right plot shows a magnified region of the relative ${\mathrm{tMRO}}_{2}$ data extracted from the green, dotted rectangle in the top-left plot. This expanded view clearly shows Mayer waves (double-sided arrow) with a period of about 10 s. Mayer waves are slow oscillations due to sympathetic regulation of vascular tone. Within this 0.1 Hz oscillation, the heartbeat is also clearly visible (arrow with dotted line) with a period of slightly <1  s. The bottom-right plot shows the absolute metabolism corresponding to the same time period. Again, the double-sided arrow represents the period of the Mayer waves, and the dotted arrow points to the heartbeat. Overall, these signals demonstrate cSFDI sensitivity to ${\mathrm{tMRO}}_{2}$ dynamics throughout the postrelease phase.

**Fig. 6:**
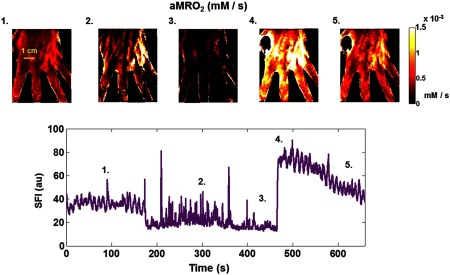
Tissue metabolic rate of oxygen consumption (${\mathrm{tMRO}}_{2}$) property maps. The images along the top are maps of ${\mathrm{tMRO}}_{2}$ in mM/s (millimoles per liter per second). The bottom plot shows the SFI obtained at 852 nm from an ROI. The numbers at the top left of each ${\mathrm{tMRO}}_{2}$ image correspond to the times in the SFI plot. Image 1 was calculated at baseline, images 2 and 3 were calculated at different times during the occlusion, and images 4 and 5 were calculated at different times following the release.

The images along the top of Fig. 6 show ${\mathrm{tMRO}}_{2}$ maps in millimoles per liter per second at different times throughout the arterial occlusion protocol. The plot beneath the images shows the same SFI time-series data presented in Fig. 6 and is provided as a reference for the ${\mathrm{tMRO}}_{2}$ image sequence. The numbers at the top left of each image correspond to the numbers in the SFI plot. Image 1 is taken from the entire baseline duration from 0 to 180 s (see Sec. 2 for details). Images 2 and 3 were calculated from the middle to end of the occlusion and correspond to 260 and 480 s, respectively. Both images 2 and 3 required a linear fit to [HHb] as described in Sec. 2. Images 4 and 5 were derived from the postrelease phase and correspond to 490 and 600 s, respectively. The ${\mathrm{tMRO}}_{2}$ images demonstrate a progressive reduction in tissue metabolic rate during the occlusion. These data also demonstrate a substantial increase in ${\mathrm{tMRO}}_{2}$ associated with the release of the occlusion that gradually decreases with repayment of the oxygen debt.

Table 1 shows the average metabolic parameters calculated for three subjects aged 29 to 46 who took part in the arterial occlusion protocol. Subject 1 underwent a 5-min occlusion, while subjects 2 and 3 underwent 3-min occlusions. The baseline, intraocclusion, and postocclusion metabolism are provided in units of millimoles of oxygen per liter per second (mM/s), and the relative metabolism is given in arbitrary units. The table provides the mean and standard deviation of each parameter calculated over 100 separate 10-pixel×10-pixel ROIs (6  mm×6  mm). The baseline metabolism is calculated from the first 30 s of the arterial occlusion, whereas the intraocclusion metabolism is calculated from the final 30 s of the arterial occlusion. For the rationale behind using the first 30 s for baseline, see Sec. 2.6. The relative metabolism is the average ratio of the postocclusion metabolism to the baseline metabolism. Because this ratio is calculated at each ROI and then averaged, it is not exactly equal to the ratio of the baseline to post-occlusion values provided in Table 1.

**Table 1:** Average metabolic calculations for three subjects. Each entry contains the mean and standard error calculated from 100 separate 10-pixel×10-pixel regions (6  mm×6  mm).

	Subject 1	Subject 2	Subject 3	Units
Baseline metabolism	$(3.5\pm 0.09)\times 10^{-4}$	$(2.7\pm 0.07)\times 10^{-4}$	$(2.9\pm .1)\times 10^{-4}$	mM/s
Intraocclusion metabolism	$(9.8\pm 1.4)\times 10^{-5}$	$(1.9\pm 0.08)\times 10^{-4}$	$(1.0\pm .09)\times 10^{-4}$	mM/s
Postocclusion metabolism	$(10\pm 1.6)\times 10^{-4}$	$(7.8\pm 1.8)\times 10^{-4}$	$(6.7\pm 0.2)\times 10^{-4}$	mM/s
Relative metabolism	4.6±0.9	3.8±0.8	3.1±0.05	a.u.

### 3.5 Rabbit Model of Cyanide Poisoning

**Fig. 7:**
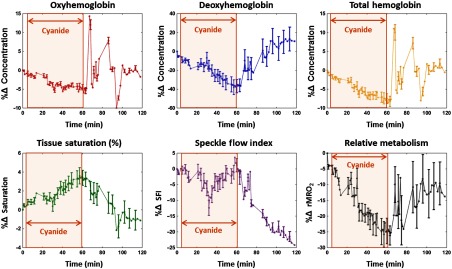
Rabbit cyanide cSFDI experimental results. Along the top from left to right, the plots contain the percent change from baseline in tissue concentrations of deoxyhemoglobin, oxyhemoglobin, and total hemoglobin, respectively. Along the bottom from left to right, the plots contain percent change in tissue saturation, SFI, and relative tissue metabolic rate of oxygen consumption (${\mathrm{rMRO}}_{2}$).

Figure 7 presents metabolic imaging data acquired from the rabbit model of cyanide poisoning. The time-series data in each plot are derived from a 1-cm×1-cm ROI within the lower right abdomen. From left to right, the top row presents plots of percent changes in deoxyhemoglobin (%ΔHHb), oxyhemoglobin ($\%\Delta {\mathrm{HbO}}_{2}$), and total hemoglobin (%ΔTHb). From left to right, the bottom row shows percent changes in tissue saturation ($\%\Delta {\mathrm{StO}}_{2}$), speckle flow index (ΔSFI), and relative metabolism ($\%\Delta {\mathrm{rMRO}}_{2}$). In each graph, the x-axis represents time in minutes. The brown shaded box outlines the duration of the cyanide infusion. The antidote is administered immediately after the cyanide infusion ends, visually depicted as the right edge of the brown, shaded box.

Deoxyhemoglobin, pictured in the top-middle panel, decreases by 35% from baseline (t=0  min) to the end of the cyanide infusion (t=60  min). After the cyanide infusion is stopped and the antidote is administered at t=60  min, deoxyhemoglobin eventually increases by 45% by the end of the experiment at t=120  min. Oxyhemoglobin, pictured in the top-left panel, decreases by 5% throughout the infusion of cyanide. Following the end of the cyanide infusion and the antidote bolus, the oxyhemoglobin begins to fluctuate erratically before returning to baseline at the end of the experiment (t=120  min). Total hemoglobin, pictured in the top-right panel, decreases by 8% during the cyanide infusion (t=60  min) and then eventually returns to baseline following the antidote (t=120  min). Tissue saturation, pictured in the bottom-left panel, increases by 3% from the beginning (t=0  min) to the end (t=60  min) of the cyanide infusion. It then decreases by 4% from the end of the infusion and the administration of the antidote (t=60  min) to the end of the experiment (t=120  min). SFI obtained from the 660-nm laser diode, pictured in the bottom-center plot of Fig. 7, remains relatively constant throughout the infusion and then decreases by 25% from the end of the infusion (t=60  min) to the end of the experiment (t=120  min). In this experiment, 660 nm was used for SFI because of better signal quality (versus 852 nm), possibly due to rabbit skin having different characteristics than human skin. Relative metabolism, pictured in the bottom-right panel, decreases by 25% from baseline (t=0  min) to the end of the infusion (t=60  min). It then increases by ∼15% from the end of the infusion/administration of the antidote (t=60  min) to the end of the experiment (t=120  min).

## 4 Discussion

The development of quantitative, noncontact, label-free methods for real-time imaging of tissue metabolism over a wide, scalable FOV would have broad impact in medical imaging and image-guided therapy. In this work, we introduce several technical innovations to achieve this goal based on the sinusoidal projection of two long-coherence-length, near-infrared (NIR) laser diodes at a fixed spatial frequency pattern ($f=0.2\;{\mathrm{mm}}^{-1}$). Using a single snapshot Fourier demodulation technique and model-based analysis of light transport in the spatial frequency domain, we simultaneously acquire the SFI while generating tissue oxy- and deoxyhemoglobin concentration maps. By combining the coregistered SFI and tissue hemoglobin parameters, we demonstrate that ${\mathrm{tMRO}}_{2}$ can be acquired at sufficiently high-speed (16  frames/s in this work) to reveal spatial and temporal dynamics in tissue metabolism, which are otherwise difficult or impossible to measure.

Dual-wavelength single snapshot cSFDI utilizes only one projection frequency per wavelength, allowing for fast measurements and simplified instrumentation. Since only a single detector is used in acquisition, there is intrinsic coregistration of all parameters, which simplifies the synthesis of tissue chromophore and SFI data into ${\mathrm{tMRO}}_{2}$. An important feature of this technique is that spatial modulation allows cSFDI to decouple tissue optical properties, i.e., absorption ($\mu_{a}$) from reduced scattering ($\mu_{s}^{\prime }$) parameters to produce more accurate quantitative tissue measurements compared with conventional unstructured illumination.^18^ Tissue $\left[{\mathrm{HbO}}_{2}\right]$ and [HHb] are then calculated directly from dual-wavelength $\mu_{a}$ values. Reduced scattering, although not explored in this work, is also an important parameter with relevance to burn wound classification and tissue structural orientation.^37^^,^^47^

Yeast-hemoglobin tissue phantom experiments validate cSFDI performance over the full range of hemoglobin saturation values, reproduce the correct sigmoidal desaturation curve, and linearly quantify [HHb] and $\left[{\mathrm{HbO}}_{2}\right]$ compared with a conventional commercial SFDI device. We note that the conventional SFDI method used for comparison employed three optical wavelengths, five spatial frequencies at each optical wavelength, and three spatial phases for each frequency. As a result, a total of 45 raw images (3×5×3) were used for each measurement. By comparison, dual-wavelength single snapshot cSFDI used only two optical wavelengths and two spatial frequencies (f=0 and $0.2\;{\mathrm{mm}}^{-1}$). Importantly, these two spatial frequencies are embedded within a single image and do not require three spatial phases, yielding a total of just two raw images per measurement, thus enabling a much higher sampling rate. Consequently, dual-wavelength cSFDI is unable to account for additional chromophores, which is why baseline optical properties were subtracted in the yeast-hemoglobin phantom study. This is due to the fact that Intralipid has a nonzero extinction coefficient. Together, the yeast-hemoglobin and hemoglobin titration experiments indicate that cSFDI has the potential to accurately measure ${\mathrm{tMRO}}_{2}$ insofar as chromophore recovery is accurate. In previous studies, we have shown that the SFI obtained using cSFDI is linear and robust with respect to volumetric flow;^38^ hence, we did not perform additional validation of flow properties in this work.

*In vivo* human arterial occlusion studies demonstrate that cSFDI can measure dynamic changes relevant to human physiology. The chromophore time-series data in Fig. 4 show sensitivity to oxygen extraction during the occlusion with increasing [HHb] and decreasing $\left[{\mathrm{HbO}}_{2}\right]$. However, total hemoglobin [HbT] remains constant, which suggests that, as expected, blood does not enter or leave the compartment during an arterial–venous occlusion. The SFI tracings also demonstrate the ability to measure physiologic changes. In Fig. 4, SFI drops immediately to a residual level (biological zero) during the occlusion and it increases immediately after the occlusion to a level substantially higher than baseline. This accurately reflects what is known to happen physiologically. At the onset of the occlusion, blood flow to the subject’s arm is instantaneously clamped off using a pneumatic cuff. When the occlusion is released, blood flow immediately spikes to a higher level than at baseline. This is further evidence that cSFDI can detect postocclusive reactive hyperemia—the increase in blood flow following occlusion caused by accumulation of metabolic byproducts. [HbT] and $\left[{\mathrm{HbO}}_{2}\right]$ also show a spike following the occlusion due to this same effect.

Integrating this information into metabolism tells a more complete story. The ${\mathrm{tMRO}}_{2}$ data show clear evidence of decreased metabolism due to ischemia during the occlusion as well as increased ${\mathrm{tMRO}}_{2}$ associated with oxygen debt repayment brought on by the release. Being able to detect this information is important for a number of reasons. In PAD patients whose limbs are subject to chronic ischemia, tissue has adaptively reduced oxygen consumption, meaning that an ischemic challenge would not be expected to affect the tissue as severely. In such situations, the oxygen debt accrued by the arterial challenge would be measurably less than in a healthy subject. In this clinical setting, cSFDI could provide a valuable means for tissue metabolic characterization for therapeutic monitoring and disease stratification. It is also important to emphasize that the ${\mathrm{tMRO}}_{2}$ data presented in Figs. 5 and 6 indicate that metabolism is not static. Figure 5 in particular shows that there is a substantial reduction in ${\mathrm{tMRO}}_{2}$ during the postrelease period in addition to large variations brought on by sympathetic fluctuations in blood pressure (Mayer waves) and the heartbeat. This reinforces the significance of real-time measurements for accurately characterizing dynamic metabolism. If the acquisition time for a single measurement were not sufficiently short, these trends would be averaged out.

Previous measurements of skin oxygen consumption using oxygen electrodes recorded an average dermal respiration rate of $1470\;\mathrm{mL}\;O_{2}\;\min^{-1}\;m^{-3}$.^48^ In comparison cSFDI yields average baseline skin oxygen consumption rates of $3.0\times 10^{-4}\;\mathrm{mM}/s$ (Table 1), which can be converted to $514\;\mathrm{mL}\;O_{2}\;\min^{-1}\;m^{-3}$ at skin temperature of 34°C. There are a number of potential reasons why these measurements differ. cSFDI is based on noncontact optical imaging of blood flow and hemoglobin, whereas Evans and Naylor^48^ utilized oxygen tension-sensitive needle-electrodes inserted into the dermis. Needle electrodes probe a finite dermal region close to the electrode tip, whereas cSFDI interrogates a relatively large tissue volume with contributions from epidermis, dermis, subcutaneous fat, and connective tissue. The presence of low oxygen consumption rate tissues, such as subcutaneous fat and connective tissue within the cSFDI measurement compartment, likely account for this discrepancy. Importantly, the 3.1- to 4.6-fold increase in cSFDI measured metabolism following 3- and 5-min occlusions, respectively, is proportional to the increasing duration of oxygen debt. This is also similar to what has been seen in studies using invasive oxygen electrodes^49^ and further supports the sensitivity and realistic performance estimates of cSFDI metabolic measurements.

Finally, experiments using a rabbit model of cyanide poisoning show that cSFDI is capable of detecting mitochondrial uncoupling brought on by inhibition of cytochrome c oxidase. During the 60-min cyanide infusion, cSFDI detected an increase in saturation of 3% and a decrease in relative metabolism of about 40%. The trend in the saturation obtained with cSFDI agrees with previous work involving DOS, which showed a 10% increase in tissue saturation under similar experimental conditions.^44^ The difference in saturation changes may be attributable to the difference in measurement volumes in that cSFDI measures superficial tissue, whereas DOS measures the underlying muscle.

The experiment also demonstrates the advantage of extracting ${\mathrm{tMRO}}_{2}$ versus tissue hemoglobin oxygen saturation (${\mathrm{StO}}_{2}$) alone. If ${\mathrm{StO}}_{2}$ were the only output, the cyanide infusion response could be interpreted incorrectly as increased tissue perfusion. This is due to the fact that, for a given tissue, increasing ${\mathrm{StO}}_{2}$ levels normally indicates reduced oxygen extraction and increased tissue blood flow. However, the SFI data alone clearly show that flow did not increase during the cyanide infusion. As a result, cSFDI measurements provide a more complete picture of the underlying physiology and reveal difficult-to-measure uncoupling of blood flow and metabolism. Because metabolic uncoupling can occur in a variety of tissue pathologic states, methods for rapidly characterizing and imaging the magnitude and spatial extent of this phenomenon are expected to have utility in clinical and translational medicine.

## Figures and Tables

**Fig. 8:**
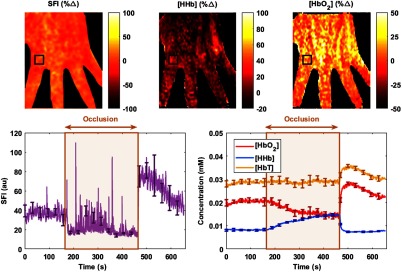
Still image from a video of dynamic changes during an arterial occlusion and reperfusion challenge. The images from left to right depict maps of percent change of SFI, [HHb], and $\left[{\mathrm{HbO}}_{2}\right]$, respectively. The bottom-left plot shows time-series SFI in arbitrary units versus time in seconds. The bottom-right plot shows time-series [HbT], $\left[{\mathrm{HbO}}_{2}\right]$, and [HHb] in yellow, red, and blue, respectively. (Video 1, QuickTime, 11.4 MB [URL: https://doi.org/10.1117/1.JBO.23.3.036013.1]).

## Appendix: Dynamic Changes During Arterial Occlusion

Figure 8 shows a still image from a video of dynamic changes during the arterial occlusion described in Sec. 3.3 and Fig. 4. From left to right, the images depict percent change from baseline (t=0  s) of SFI, [HHb], and $\left[{\mathrm{HbO}}_{2}\right]$, respectively. The black rectangle in each image defines the 1-cm×1-cm ROI from which the time-series data were obtained. The bottom-left plot shows SFI at 852 nm arbitrary units versus time in seconds. The plot on the bottom-right shows [HbT], $\left[{\mathrm{HbO}}_{2}\right]$, and [HHb] in yellow, red, and blue, respectively. The brown, shaded region in both plots outlines the duration of the 5-min arterial occlusion. The error bars overlaid on each plot depict the standard deviation of the values contained within the ROI. The video has been sped up by a factor 20 to emphasize dynamic parameter changes. Note that these values were calculated from the original data, not from the percent change presented in this figure. For the original images, refer to Fig. 4.
